# Impact of Critical Event Checklists on Anaesthetist Performance in Simulated Operating Theatre Emergencies

**DOI:** 10.7759/cureus.4376

**Published:** 2019-04-03

**Authors:** Asad Siddiqui, Elaine Ng, Claire Burrows, Duncan McLuckie, Tobias Everett

**Affiliations:** 1 Anesthesia, The Hospital for Sick Children, University of Toronto, Toronto, CAN; 2 Anesthesia and Pain Medicine, The Hospital for Sick Children, University of Toronto, Toronto, CAN; 3 Anesthesia, Western Health, Melbourne, AUS; 4 Anesthesia, Alberta Children’s Hospital, Calgary, CAN

**Keywords:** critical event checklist, cognitive aid, simulation, critical events

## Abstract

Introduction

Crises in the operating theatre during a paediatric case are rare with the incidence of anesthesia-related cardiac arrest in non-cardiac patients being 1.4/10,000. In order to address this, the Society for Pediatric Anesthesia (SPA) developed cognitive aids (CAs) in the form of Critical Event Checklists (SPA CECs). Several studies have demonstrated the benefit of CAs in improving performance of critical tasks. Despite the presence of CAs, individuals often do not use the aids consistently. The objective of our study was to investigate whether the presence of SPA CECs, and orientation to these tools, improve the performance of trainees during simulated critical events.

Methods

With local Research Ethics Board (REB) approval we used a randomized, 2 x 2 factorial design. The first randomization was the participant orientation to the SPA CECs (e-module vs. didactic). The second randomization assigned participants to complete the simulations with or without SPA CECs available. The simulations were videoed and rated by two raters using a scenario-specific checklist and global rating scale (GRS).

Results

We conducted 78 simulations. The SPA CEC was used in 17.9% of scenarios. The SPA CEC was used in 44.8% of diagnosis-based scenarios and only 2.0% of generic problem-based scenarios. Participants’ performance was superior with the SPA CEC present (GRS mean 3 [SD 1.27]) than without the SPA CEC available (GRS mean 2.43 [SD 0.89]) (p = 0.048).

Conclusion

Overall, we showed that uptake of the SPA CECs is poor. We also demonstrated that when the SPA CECs are utilized, they enhance the performance of trainees in simulated operating room (OR) critical events.

## Introduction

Crises in the operating theatre during a paediatric case are fortunately rare with data from the Paediatric Perioperative Cardiac Arrest registry suggesting that the anesthesia-related incidence of cardiac arrests in non-cardiac patients is 1.4 in 10,000 [1]. Management strategies of emergency situations are rapidly forgotten with the retention of advanced life support knowledge diminishing significantly within as little as six months post-certification [2] and passing candidates being unable to pass a re-test as few as four months later [3].

In order to counter this knowledge attrition, there has been an increased interest in the implementation of peri-operative cognitive aids. A sub-set of cognitive aids (CAs) are checklists that set out a list of essential action points to be completed during an adverse event. For paediatric operating theatre emergencies, the Society for Pediatric Anesthesia (SPA) has developed a set of Critical Event Checklists (SPA CECs) that are available online and as a smartphone app (Pedi Crisis, Version 2.0, Society for Pediatric Anesthesia, Richmond, VA, USA) [4, 5].

Several studies have demonstrated the benefit of CAs on various outcome measures including adherence to guidelines, time to complete essential tasks and overall performance in crisis situations [6, 7-11]. Through the use of CAs, teams also demonstrate an improvement in anaesthetists non-technical skills (ANTS) [10, 12, 13]. However, CAs can only be applied effectively in the context of sound clinical knowledge and skills to avoid inappropriate use of CAs. For example, Nelson et al. demonstrated that 26% of paediatrics trainees provided inappropriate management of simulated cardiopulmonary arrest when using CAs after choosing the wrong treatment algorithm [14]. Everett et al. demonstrated that 25% of teams chose an incorrect CA when managing critical events in a simulated daycase operating theatre setting [15]. Furthermore, Bould et al. demonstrated that the uptake of CAs is poor with only 26.7% of participants in a neonatal resuscitation simulation using the tool frequently when it was available [3]. This is in contrast to survey findings that almost 80% of individuals believe that CAs improve care in critical situations and would use a CA if it were available to them [16].

The minimal or incorrect use of CAs, despite their presence in simulated crises, can potentially be attributed to unfamiliarity and lack of orientation to the CAs [17-19]. In a recent editorial, Marshall emphasizes the importance of education regarding CAs prior to their testing and that future research should investigate implementation strategies that maximize uptake [20]. In a cross-sectional study identifying factors associated with use of CAs, providing adequate time for training in the use of CAs was identified as a factor towards its successful implementation [21].

In this study, our primary aim was to determine the uptake of SPA CECs in a simulated operating theatre emergency and the performance enhancement conferred by using the SPA CEC. We investigated interventions surrounding increasing uptake of the SPA CEC through different modalities of orientation to the tool (didactic vs. e-module) as well as visible availability of the SPA CEC in the simulated operating theatre. Our primary hypothesis was that e-module orientation and the visible availability of the SPA CEC would translate to greatest uptake of the SPA CEC. Our secondary hypothesis was that use of the SPA CEC would confer a measurable performance enhancement for the practitioner involved.

## Materials and methods

Population

With local Research Ethics Board (REB) approval (REB 1000051296), senior anaesthesia trainees at a single university-affiliated tertiary level paediatric hospital were recruited to be enrolled in the study. Written informed consent was obtained from participants in regards to participating in the study as well as for videotaping of the simulation.

Sample size calculation

Available data on the impact of critical event checklists in similar contexts [3, 6] led us to expect a 30% performance enhancement conferred by the SPA CECs. Allowing for a two-sided α error of 0.05, a power of 80% (β = 0.2), and repeated measures per participant, our target recruitment was 24 participants.

Study design

We used a randomized, 2 x 2 factorial design (Table 1), with an interest in the combination intervention as well as individual interventions.

**Table 1:** 2 x 2 factorial design. ^a ^SPA CECs: Society for Paediatric Anesthesia Critical Event Checklists

		Availability of SPA CECs		
		SPA CEC available	SPA CEC unavailable	Total
Orientation to SPA CECs^a^	Orientation A (Didactic)	Group A (n = 24)	Group B (n = 16)	40
	Orientation B (E-Module)	Group C (n = 16)	Group D (n = 22)	38
	Total	40	38	78

Consent was sought in person by a study investigator who had no influence over the program evaluation or career progression of the participants. After this, the first level of randomization and the first explanatory variable was the mode of participant orientation to the SPA CECs. Participants were oriented either by a didactic method or by an e-module. The e-module was constructed using the Articulate Storyline software (Articulate, NY, USA). The duration and content of each mode of orientation was identical in that the didactic session was tightly scripted to follow the same framework as the e-module. The second level of randomization divided participants into groups completing their simulation scenarios with or without the SPA CECs available (a printed binder in the operating room). Therefore, the second explanatory variable was the visible availability of the SPA CECs to participants. We used online randomization software for both levels of randomization.

Table 1 shows the four groups generated. These scenarios were randomly selected from the Managing Emergencies in Paediatric Anaesthesia (MEPA) course content. The MEPA course is an international simulation-based training course in paediatric anaesthesia [22-24]. The course content and assessment tools associated have been subjected to peer review and extensive investigations of their validity [15,23]. The assessment tools involve a scenario-specific performance checklist which quantifies medical management and a Global Rating Scale (GRS) which allows the rater to score overall performance (from novice to expert, including consideration of non-technical skills), and prompts the rater to consider if the candidate is performing at the level of a consultant anaesthetist. The MEPA GRS is a six-point scale where a score of 4-6 is reserved for practitioners performing at the level of a consultant anaesthetist. The MEPA scenarios used in this study were:

 1. Laryngospasm

 2. Malignant Hyperthermia (MH)

 3. Local Anaesthetic Systemic Toxicity (LAST)

 4. Anaphylaxis

 5. Retained Throat Pack

 6. Hypovolaemia

 7. Equipment Failure (obstructed breathing circuit)

Participants were randomly assigned using an electronic random number generator to participate in three or four of the seven scenarios (over two days per candidate). They were randomly assigned to avoid the possibility that particular combinations of scenarios were more or less challenging or more or less likely to induce uptake of the SPA CECs.

A SimBaby mannequin (Laerdal Medical, Stavanger, Norway) was used for the simulation and the SimCapture Ultraportable (B-Line Medical, Washington, USA) was used to video-record the sessions. The simulation was setup in a consistent manner between simulations with consistent setup of the mannequin, equipment and cameras. The video-recordings of the simulation sessions were encrypted and stored in a secure location.

Two expert raters received rigorous standardized training in using the MEPA GRS and checklist. The raters were blinded to the study hypothesis and rated independently. Inter-observer agreement was expressed in terms of intra-class correlation coefficients (ICCs) and described according to the scheme suggested by Cicchetti [25], where an ICC < 0.4 is considered poor agreement, 0.4-0.59 is fair, 0.6-0.74 is good and >0.75 is considered excellent agreement. The ICC was calculated at two points. First, the ICC was assessed after a sample of 21 videos (three of each scenario) to ensure that there was acceptable reliability and to determine whether re-training was necessary. Second, the ICC was calculated after completion of rating of all 78 videos by both raters.

Statistical analysis

The primary outcome of the study was whether participants used the SPA CEC to assist them in their management of the operating theatre crisis. Given the 2 x 2 factorial design, a two-way ANOVA was used to analyze the impact of the two independent variables (mode of orientation and availability of CEC). The main effects from each of the two factors as well as the interaction effect of the two factors was derived. A multivariable model was used to analyze the dependent variables of (1) uptake of cognitive aids (2) performance on simulation scenarios. A significant difference was defined by a two-tailed p-value less than 0.05.

With respect to uptake of SPA CECs, we assessed the impact of diagnosis-based checklists (e.g., local anaesthesia systemic toxicity or malignant hyperthermia) vs. generic event checklists (e.g., non-specific hypoxemia or hypotension) using classical hypothesis testing with a significant difference defined by a p-value less than 0.05.

Performance enhancement conferred by use of SPA CECs was analyzed using an independent-samples t-test to compare means with a two-tailed significance level defined as a p-value less than 0.05. All statistical analysis was performed using SPSS version 23 (IBM, Armonk, NY, USA).

## Results

Twenty-five senior anaesthesia trainees (postgraduate year four or higher) engaged in 78 simulation encounters, distributed among the four groups as shown in Table 1. The proportion of encounters in which participants used the SPA CECs is presented in Table 2. The overall uptake of the SPA CECs in the simulated critical events was 17.9%. The uptake of the SPA CECs was not significantly different whether participants received an e-module or a didactic orientation to the SPA CEC (16% vs. 20%, respectively, p = 0.690). The proportion of encounters where the SPA CEC was used did not differ significantly whether the SPA CEC was available (in clear view) or not (20% vs. 16%, respectively, p = 0.690). There was no interaction effect between the two explanatory variables (p = 0.867).

**Table 2:** Uptake of the SPA CEC cognitive aid based on mode of orientation, availability of SPA CEC cognitive aid and type of cognitive aid. ^a ^SPA CECs: Society for Paediatric Anesthesia Critical Event Checklists

	Number of encounters SPA CEC^a^ used (%)	P-value
Didactic orientation (n = 40)	8 (20%)	p = 0.690
E-module orientation (n = 38)	6 (16%)	
SPA CEC^a^ available (n = 40)	8 (20%)	p = 0.690
SPA CEC^a^ unavailable (n = 38)	6 (16%)	
Diagnosis-based SPA CEC^a^ (n = 29)	13 (45%)	p < 0.010
Problem-based SPA CEC^a^ (n = 49)	1 (2%)	

The nature of the scenario and thus the type of SPA CEC used did influence uptake. We observed significantly greater uptake of the SPA CEC in scenarios that required a diagnosis-based SPA CEC than ones which required the use of a generic event (altered-physiology) SPA CEC. Where there was a specific diagnosis (e.g., MH, LAST, anaphylaxis) there was a 45% uptake of the SPA CECs as opposed to 2% of generic event scenarios (e.g., hypoxemia, hypotension) (p < 0.010).

All 78 simulation encounters were evaluated by two trained independent raters using the MEPA performance checklist and GRS [23]. After the first 21 videos were rated, the ICC was 0.828 for the GRS and 0.917 for the performance checklist indicating excellent inter-rater reliability and no need for re-calibration. All 78 videos were rated by both raters. Their final inter-rater reliability was also excellent (ICC = 0.764 for the GRS and ICC = 0.801 for the performance checklist).

The influence of the SPA CEC orientation and the SPA CEC use on clinical performance is shown in Table 3. The mode of orientation to the SPA CEC did not produce a difference in performance scores. Considering the impact of using the SPA CEC, we showed a significantly superior clinical performance when participants did use the SPA CEC than when they did not [GRS 3.0 with the SPA CEC vs. GRS 2.4 without the SPA CEC (p = 0.048)]. The other rating tool showed a trend suggestive of superior performance with the use of the SPA CEC which did not reach significance [52.1% with a CEC vs. 44.5% without a CEC (p = 0.065)].

**Table 3:** Clinical performance as influenced by mode of orientation to SPA CEC and impact of using SPA CEC. ^a ^SPA CEC: Society for Paediatric Anesthesia Critical Event Checklists ^b ^GRS: Global Rating Scale

	GRS^b^ (1-6) Mean ± SD	P-value	Performance Checklist % Mean ± SD	P-value
Didactic orientation (n = 40)	2.5 ± 1.0	p = 0.891	45.4 ± 15.2	p = 0.779
E-module orientation (n = 38)	2.5 ± 1.0		46.3 ± 12.8	
SPA CEC^a^ used in management (n = 14)	3.0 ± 1.3	p = 0.048	52.1 ± 18.8	p = 0.065
SPA CEC^a^ not used in management (n = 64)	2.4 ± 0.9		44.5 ± 12.5	

## Discussion

In the field of cognitive aid implementation, there is a disconnect between practitioners stated desire for the tool and their uptake in relevant circumstances. Orientation and education regarding new CAs is an important component of their implementation [20,21]. We aimed to orientate and train trainees to use the SPA CECs by delivering standardized, scripted content either packaged as an interactive e-module or as a didactic presentation. We failed to show that either mode of orientation had a superior impact on the SPA CEC uptake or clinical performance. In fact, it could be argued that both modes of orientation were similarly poor, because the overall uptake of the SPA CECs was only 18%. A simple explanation was that on arrival at a new institution, trainees are given a diverse variety of orientation sessions, such that one extra e-module or one extra didactic would be subsumed among the volume of other material delivered around the same time. This gives us pause to consider the efficacy of welcome orientations with all the material delivered at once at the start of a rotation. Furthermore, Alidina et al. describe a multifactorial process contributing towards the use of CAs in the OR; therefore, factors such as organizational contexts and leadership supports may also be necessary, in addition to appropriate orientation and education, to improve uptake of CAs [21].

Another reason that orientation mode did not influence uptake may be the use of a purely e-module or didactic orientation as opposed to a blended model. In 2005, Childs et al. conducted a systematic review assessing the barriers of e-learning in the health field. One of the potential problems that they identified was the need for a face-to-face component to confer effective learning [26]. In our study, participants in the e-module orientation group received no face-to-face component to their orientation which may have diminished the potential learning and explain the lack of impact on SPA CEC uptake. It is suggested that simulation-based orientation leads to significant increases in self-reported familiarity and willingness to use cognitive aids [27]. A logical extension of our research is to investigate if a simulation-based orientation to the SPA CECs influences their downstream use by practitioners, either in the simulated or real environment.

We found that in cases where the SPA CECs were used there was a significant preference for the diagnosis-based checklists. When participants were faced with an evolving generic situation, the foundation of which was still unclear (e.g., hypoxemia or hypotension), they would not reach for the relevant generic event SPA CEC, despite their availability. It was far more common for participants to use the SPA CECs in specific diagnosis situations. We were not surprised by this finding. A checklist is not a substitute for knowledge, judgement, critical thinking, or clinical decision-making. The bullet-point format of the SPA CEC may be best suited when there is a recognized list of necessary actions to take in a definable emergency. We further reflected that although the SPA CECs once deployed in the real operating theatre would be rarely used, there will be a subset within them that will likely never be used.

Our study showed that clinical performance was enhanced by the use of a CEC, with a mean difference in GRS of 0.57 (out of six). In previous research from our group we have shown a correlation between accumulated months spent in anesthesiology training and superior performance on the MEPA GRS [28]. The gradient of that linear relationship is that on average an increase in practitioner score of 0.57 occurs over approximately 20 months of anesthesiology training (Abstract: Everett T, Ng E, Kulkarni P, Borges B, Letal M, Fleming M, Bould MD. Simulation-Based Assessment: A Multicenter Validation Study. CAS Conference; June, 2016). If we can boost anesthesiologist performance in a crisis using cognitive aids, then this makes a good justification for the integration of the SPA CECs into the workplace and training programs.

Prior studies have demonstrated that when individuals are asked to use checklists, at times they select an inappropriate checklist and hinder their performance [14]. We did not see this phenomenon in our study - although uptake was low, where the SPA CECs were used they were correctly chosen and had a positive impact on performance. This may have been because as checklist use was optional, the participants only reached for it once they were confident that they knew the diagnosis, could match the SPA CEC to it and it would yield useful prompts. We noted that the GRS scores (whether or not the SPA CEC was used) were largely below the passing score for a new consultant anaesthetist (GRS of four). This is acceptable because the participants were senior trainees, but is in conflict with our other research which showed that senior trainees and new consultants performed similarly in these scenarios (Abstract: Everett T, 2016). This caused us to reflect on whether imposing checklists, with the associated processes had been a distraction and had been detrimental to the clinical performance of the participants. Due to a number of potential confounders between study populations, we were not in a position to examine this phenomenon statistically.

Strengths and limitations

One of the strengths of our study was the generalizability to real practice as participants were not obliged to use CAs despite their availability. This was especially important in our study given that one of our outcomes was uptake of CAs, and gives us useful information about the real-life effectiveness of the SPA CECs.

A limitation of our study is that although the SPA CECs are provided by the Society for Pediatric Anesthesia, they are meant for whole-team use, rather than just the anaesthetist. In the current study we were examining their uptake and impact during uniprofessional simulation training. In other work from our group, we have evaluated their impact in in-situ interprofessional team training, and continue to investigate this question.

Another limitation of our study was that we did not analyze retention at a later stage (i.e., six months) surrounding the impact of our independent variables. For example, our study cannot detect whether our modes of orientation had a delayed impact on uptake of CAs.

Future areas of research

We did not show a significant difference between e-module and didactic orientation to the uptake of CAs. However, determining a means of effective orientation and increasing familiarity with CAs is important. There is literature suggesting the potential effectiveness of simulation-based orientation and repetitive practice [27,28]. Therefore, one novel means of orienting trainees to CAs may be through repetitive practice using simulation. This provides an area for future research to help increase the uptake of CAs.

Our study also demonstrated that the uptake of CAs was significantly higher with diagnosis-based checklists as opposed to generic event checklists. In order to explore this further, additional studies need to be conducted investigating the specific impact of CAs in diagnosis-based and generic event scenarios. For example, if participants are required to use a generic event cognitive aid in a scenario that they normally would not (i.e., a hypoxia checklist), does it actually impair their performance? Furthermore, a potential area of future research includes using a qualitative approach to further understand why and when individuals use CAs.

## Conclusions

We demonstrated that uptake of CAs is poor despite formalized orientation, that certain types of CAs are used more frequently than others, and that when individuals did use the SPA CEC, they performed better than participants that did not use the SPA CEC. Further research needs to be conducted surrounding novel means of orientation and education surrounding CAs as they have important implications for patient safety and medical education.
